# Characterization of platelet-related genes and constructing signature combined with immune-related genes for predicting outcomes and immunotherapy response in lung squamous cell carcinoma

**DOI:** 10.18632/aging.204886

**Published:** 2023-07-20

**Authors:** Siyi Zhao, Han Gong, Wenhua Liang

**Affiliations:** 1 Department of Thoracic Surgery and Oncology, The First Affiliated Hospital of Guangzhou Medical University and Guangzhou Institute of Respiratory Disease and China State Key Laboratory of Respiratory Disease and National Clinical Research Center for Respiratory Disease, Guangzhou, China; 2 Department of Clinical Medicine, The First Clinical Medical School of Guangzhou Medical University, Guangzhou, China; 3 Molecular Biology Research Center and Center for Medical Genetics, School of Life Sciences, Central South University, Changsha, Hunan, China

**Keywords:** lung squamous cell carcinoma, platelet-related genes, prognosis, immunity, tumor microenvironments

## Abstract

Lung squamous cell carcinoma (LUSC) is a highly malignant subtype of non-small cell lung cancer with poor prognosis. Platelets are known to play a critical role in cancer development and progression, and recent studies suggest that they can also regulate immune response in tumors. However, the relationship between platelet-related genes (PRGs) and LUSC prognosis and tumor microenvironments remains unclear. In this study, we used multiple bioinformatics algorithms to identify 25 dysregulated PRGs that were significantly associated with LUSC prognosis. We found that PRGs were involved in multiple biological processes, particularly in the tumor microenvironment, and that platelet-related scores (PRS) were a risk factor. Additionally, we established a 6-gene prognostic signature combining PRGs and immune-related genes that accurately predicted outcomes and immunotherapy efficacy in LUSC patients. Our study provides a comprehensive analysis of the biological functions and potential therapeutic targets of PRGs in LUSC, which may inform the development of new treatments for this disease.

## INTRODUCTION

Lung cancer is a significant public health problem and remains one of the leading causes of cancer-related deaths worldwide [1]. Among the different subtypes of non-small cell lung cancer (NSCLC), lung squamous cell carcinoma (LUSC) has one of the poorest prognoses and highest mortality rates [2], accounting for the majority of all lung cancer cases. Despite advances in medical treatments for LUSC, including surgery, chemotherapy, and radiation therapy, the 5-year survival rate for LUSC patients remains alarmingly low, at below 30% [3]. Recently, immunotherapy has emerged as a promising treatment option for advanced-stage LUSC and has shown potential in improving patient outcomes [4, 5]. However, patient response to immunotherapy is highly variable, and not all patients benefit equally from this treatment [6]. This variability in response highlights the need for new biomarkers to predict patient response and to inform the development of personalized treatment strategies.

Platelets play a significant role in cancer development and progression, and recent studies have suggested that they may have a more complex role in cancer beyond just blood clotting [7, 8]. Platelets have been shown to be involved in the regulation of tumor angiogenesis, immune evasion, and the promotion of tumor cell proliferation [9–11]. The crosstalk between cancer cells and platelets plays a crucial role in the malignant progression of tumors. Tumor cells can activate platelets, and in turn, platelet activation can promote tumor growth and metastasis [12, 13]. Additionally, platelets have been shown to regulate immune response in tumors, affecting the efficacy of immunotherapy [14, 15]. Multiple cytokines (such as VEGF, PDGF, IL-6) secreted by platelets contribute to the formation of an immunosuppressive tumor microenvironment and can reduce the ability of NK cells to recognize tumor cells. Interestingly, there are also studies that use platelets as a biological carrier for encapsulating drugs to enhance anti-tumor immunity [16, 17]. Current research on platelets in lung cancer primarily focuses on the prognostic value of platelet counts or the platelet-to-lymphocyte ratio during clinical investigations [18–21]. However, the association of platelet-related genes (PRGs) with prognosis in LUSC is unclear.

At present, in multiple types of tumors, platelet-related genes have shown good efficacy in patient molecular subtyping and prognosis guidance, including liver cancer, esophageal cancer, and colorectal cancer [22–24]. These findings have led to increased interest in the potential of platelet-related genes as prognostic biomarkers for prognosis and immunotherapy response in LUSC. The study of platelet-related genes in LUSC has the potential to provide new insights into the biology of this disease and inform the development of personalized treatment strategies, including immunotherapy.

The aim of this study was to characterize the role of platelet-related genes (PRGs) in prognosis and tumor microenvironments, with a specific focus on establishing a gene signature for predicting immunotherapy response in LUSC patients. To achieve this, we conducted a comprehensive analysis of public gene expression datasets from LUSC patients and implemented various bioinformatics tools to identify genes related to platelets and immunity that were significantly associated with LUSC prognosis. Finally, we established a 6-gene signature based on these genes, which we validated using multiple independent datasets. The results of this study have the potential to provide a new tool for improving the prognosis of LUSC patients and to inform the development of personalized treatment strategies.

## MATERIALS AND METHODS

### Data collection and preprocessing

After screening for incomplete follow-up information and paraffin tissue samples, we retrieved transcriptome expression profiles of 489 LUSC patients and corresponding clinicopathological features from the TCGA-LUSC cohort. Simple nucleotide variations files of each patient in the format of “maf” were downloaded from TCGA portal (TCGA-LUSC project) and merged by performing the maftools package [25]. Through the GEO database, we obtained six lung cancer cohorts (GSE3141, GSE12472, GSE30219, GSE157011, GSE138682, and GSE149507) and three immunotherapy cohorts (GSE78220, GSE176307, and GSE135222). Similarly, the scRNA-seq dataset E-MTAB-6149 was obtained from the EMBL-EBI portal (https://www.ebi.ac.uk/). Referring to previous literature, RNA-Seq data were normalized to FPKM [26]. The bulk RNA expression of any genes that were zero in at least 50% of the samples was removed. The platelet-related genes (PRGs) were obtained from previous literature [27, 28], and the immune-related genes (IRGs) were obtained from the Gene List module of the ImmPort portal (https://www.immport.org/home). The LUSC patients lacking information on survival status or time less than 30 days was excluded. The patient sample information included in this study is shown in Supplementary Table 1.

### Identification of PRGs-related clusters

The “limma” package (version 3.52.4) [29] was implemented to identify dysregulated genes, and by intersecting with the PRGs, we obtained 112 PRGs according to |log2FC| > 1 and adjusted *p*-value < 0.01. Based on the expression of these PRGs and follow-up information from LUSC patients, we conducted the univariate Cox regression method to screen PRGs with prognostic value. The PRGs with *p*-value < 0.05 were obtained for further clustering analysis. We performed the “ConsensusClusterPlus” package (version 1.58.0) [30] to classify LUSC specimens into two molecular subgroups related to PRGs, using the parameters of clusterAlg = “km”, distance = “spearman” and reps = “100”. We implemented the principal component analysis (PCA) algorithm for visualizing the distribution of two PRGs-related subtypes. Kaplan-Meier product limit analysis were employed to compare the overall survival (OS) of the two PRGs-related subtypes.

### Platelet score calculation and biological processes quantification

We utilized the “Gene set variation analysis (GSVA)” package [31] to calculate the platelet score (PRS) and determine the activity levels of biological gene sets from the MSigDB database (downloaded on December 26, 2022) for each individual patient diagnosed with LUSC. The “limma” package was employed to investigate the differences in the pathway activity of distinct subtypes, and only candidates with a *p*-value < 0.001 were selected. We used the heatmap function of the “pheatmap” package (version 1.0.11) to visualize.

### Tumor microenvironment analysis

To quantify the infiltration levels of immune cell types in the tumor microenvironment (TME), we performed the “ESTIMATE” [32] and “CIBERSORT” [33] analyses on LUSC patients with default parameters. These tools were based on gene expression profiles of various immune cell types and utilized the relative expression levels of genes specific to each immune cell type to estimate the infiltration levels of different types of immune cells in the TME. These tools have been extensively used in previous similar studies [34–36].

To investigate the relationship between the established gene signature and TME, we performed the Spearman correlation analysis between the risk scores and the infiltration levels of TME. The significance of the correlations was determined using a two-tailed Student’s *t*-test.

### Identification of immune-related genes significantly associated with PRS

Weighted gene co-expression network analysis (WGCNA) [37] algorithm was employed to identify modules of co-expressed genes and to identify hub immune-related genes (IRGs) that were significantly associated with PRS. In detail, we chose the top 5000 genes with the biggest median absolute deviation and decided the optimal “powerEstimate” value was 6. The other settings were by default. We obtained 10 co-expressed modules and found that the magenta modules had the highest correlation with PRS.

### Establishment of the 6-gene signature

By intersecting IRGs and the magenta modules, we obtained 19 hub genes related to immunity and platelets. We employed the univariate Cox analysis to explore the association between these genes and prognosis and identified 11 genes with *p*-value < 0.05. To predict better the prognosis of LUSC patients, we constructed a 6-gene risk signature using a multivariate Cox regression model. The final risk score of each LUSC patient was calculated using the following specific formula:


$$Riskscore=\sum_{i=\text{1}}^{n}Coef_{i}\;\text{×}\;Exp_{i}$$

where *i* represents one of 6 genes (ARRB1, DGKA, FGG, EHD1, MMRN1 and DOCK9).

The LUSC patients were dichotomized into high- and low-risk subgroups based on the median risk scores. The gene signature was validated using the three external datasets of LUSC patients. The accuracy of the gene signature in predicting the prognosis of LUSC patients was evaluated using the receiver operating characteristic (ROC) curve. We also conducted the Univ- and Multiv-Cox methods to access the independence of the 6-gene signature and other clinical features.

### scRNA-seq analysis

We conducted single-cell data analysis using the “Seurat” package (version 4.3.0) [38]. First, we standardized the scRNA matrix and identified 600 highly variable genes for PCA analysis. Based on the standard deviation of PC changes and previous literature [39], we selected PC 20 and a resolution of 1.0 to cluster the LUSC cells and obtained 34 cell clusters. According to the canonical marker genes, we finally confirmed 11 cell types and visualized these cells by Uniform Manifold Approximation and Projection (UMAP). The AddModuleScore procedure was executed to estimate the PRS for different cell types.

### Prediction of anti-tumor drugs sensitivity

By importing the pharmacogenomic information in GDSC2 database as training dataset, we conducted the oncoPredict package (version 0.2) to access the susceptibility of about 200 anti-tumor drugs for each LUSC patient.

### Statistical analysis

Statistical analysis and visualization were performed using the R software (version 4.2.1). Unless otherwise stated, Wilcoxon sum test was used to compare the differences between distinct LUSC subtypes. Kaplan–Meier survival curves and the log-rank test were used to compare the differences in survival time. The *p*-value < 0.05 was considered statistically significant. We used the Bonferroni method to execute adjustments for multiple comparisons.

### Availability of data and material

mRNA expression matrix and follow-up information are downloaded from the TCGA and GEO. Further results or code inquiries can be directed to the corresponding author.

## RESULTS

### Identification of PRGs with prognostic values

**Figure 1:**
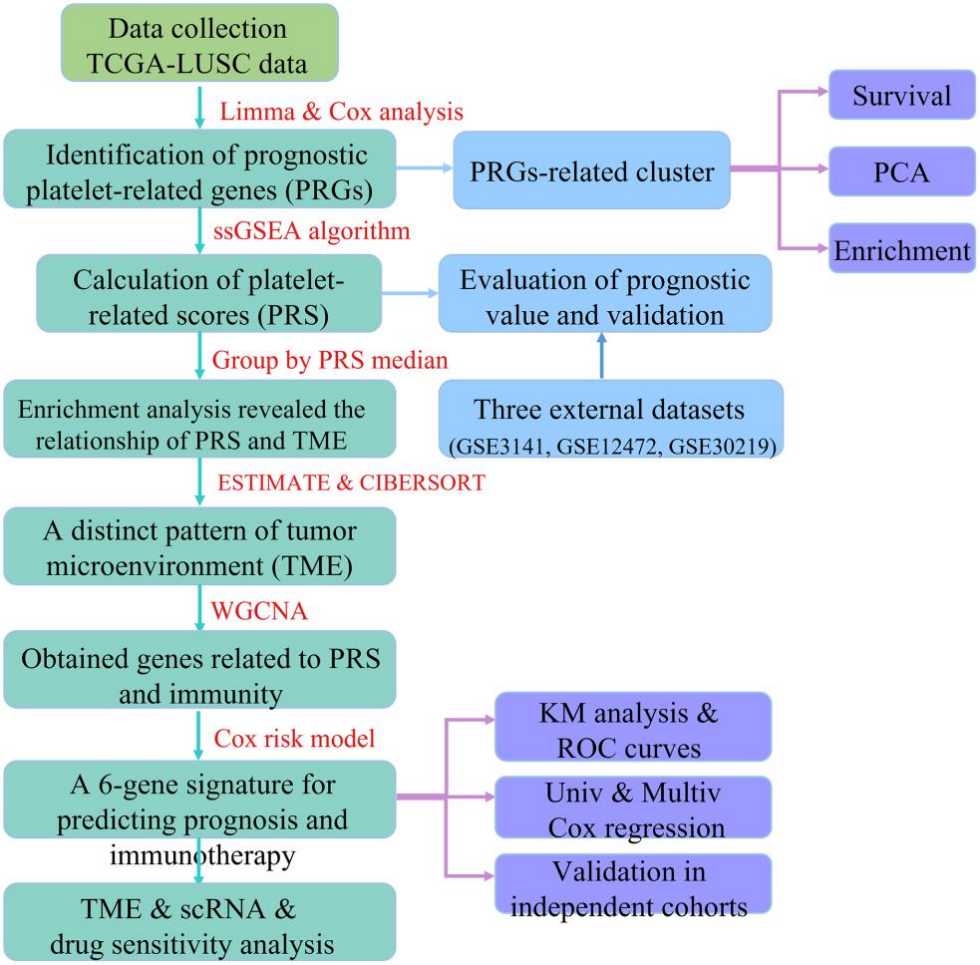
The flow chart of this study.

**Figure 2:**
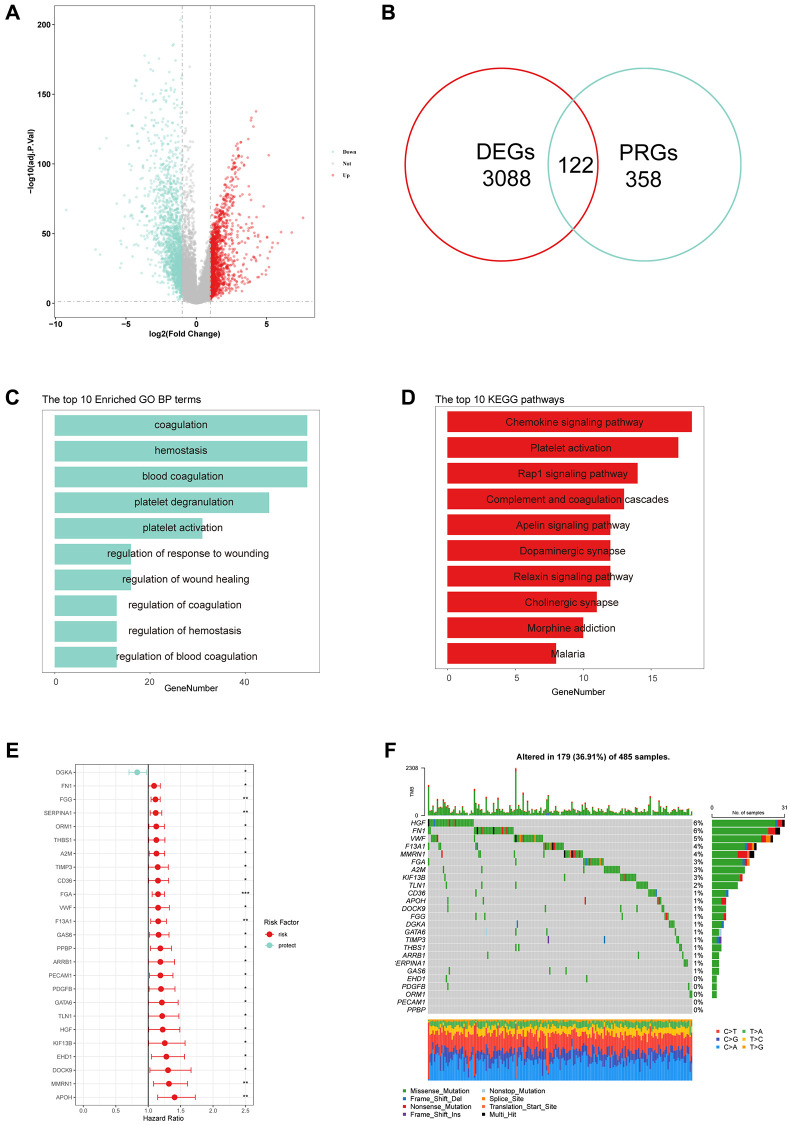
**Identification of prognostic platelet-related genes (PRGs).** (**A**) Differentially expressed genes (DEGs) in LUSC tissues compared to normal controls. (**B**) Venn plots showed that 122 common genes in DEGs and PRGs. (**C**, **D**) The top 10 results of GO and KEGG enrichment based on 122 PRGs. (**E**) Hazard ratios (HR) forest plot of 25 PRGs with prognostic values. (Red: risk factors, Blue: protective factors, ^*^p < 0.05, ^**^P < 0.01 and ^***^P < 0.001). (**F**) The waterfall plots of 25 PRGs in 485 LUSC patients.

The overall design of this study is depicted in the flow chart (Figure 1). To explore the expression of PRGs between LUSC tissues and normal controls, we first performed the “limma” package to identify dysregulated genes, and by intersecting with the PRGs, we obtained 112 PRGs (Figure 2A, 2B). The GO analysis result showed that the PRGs were involved in platelet-related pathway, such as coagulation, hemostasis and platelet degranulation (Figure 2C). The KEGG analysis result also showed that the platelet activation pathway was significantly enriched (Figure 2D). By performing univariate Cox analysis, we identified a total of 25 PRGs that were significantly associated with LUSC prognosis for further analysis (Figure 2E). Additionally, we analyzed the expression of these 25 PRGs in two external datasets and demonstrated significant dysregulation in lung cancer (Supplementary Figure 1). We further explored the frequency of gene mutation for the 25 PRGs, and found that the top 3 of most mutated genes were HGF, FN1, and VWF (Figure 2F).

### Molecular subtypes related to PRGs in LUSC

**Figure 3:**
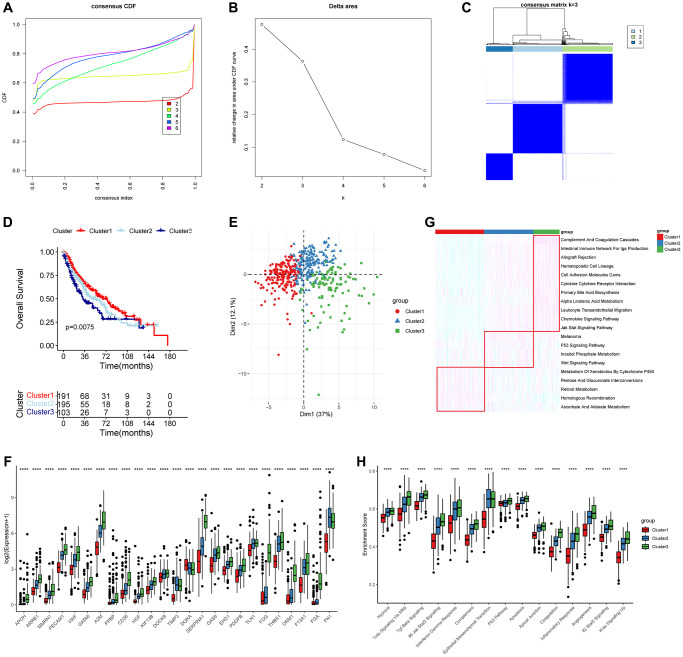
**Molecular subtypes based on 25 prognostic PRGs and biological function analysis.** (**A**–**C**) Consensus curves and heatmap when *k* = 3. (**D**) Overall survival (OS) analysis for the three distinct LUSC clusters. (**E**) PCA analysis showed that the distributions of three clusters. (**F**) Boxplot showed the expression of 25 PRGs in the three clusters (Wilcoxon test). (**G**, **H**) The significantly different KEGG and HALLMARK genesets in the three clusters using ssGSEA analysis. ^***^P < 0.01 and ^****^P < 0.0001.

We performed a consensus clustering algorithm on LUSC patients based on the expression of prognostic PRGs. According to the consensus variation curve and clustering heatmap, we determined that the best classification effect for LUSC patients was achieved when *k* = 3 (Figure 3A–3C). The KM curve showed that the cluster 3 tended to have worst clinical outcomes (log-rank test, *p* = 0.0075) (Figure 3D) and the PCA plot suggested that the three PRGs-related subtypes had significantly different distributions (Figure 3E). The boxplot illustrated that the expression of all PRGs, excluding the prognostic protective factor DGKA, was lowest in the cluster 1 (Figure 3F). The heatmap showed significant differences in biological behaviors among the three subtypes (Figure 3G). Pathways such as complement and coagulation cascades and intestinal immune network for IgA production were significantly enriched in cluster 1, while the P53 and Wnt signaling pathways were significantly enriched in cluster 2, and the homologous recombination repair and retinol metabolism were significantly enriched in cluster 3. Further analysis indicated that the activity of 50 hallmark gene sets was significantly higher in cluster 3 (Figure 3H). These findings suggested that PRGs played a role in oncogenic pathways and contributed to the development and progression of cancer, resulting in unfavorable outcomes for LUSC patients.

### Platelet scores as a prognostic risk factor of LUSC Patients

**Figure 4:**
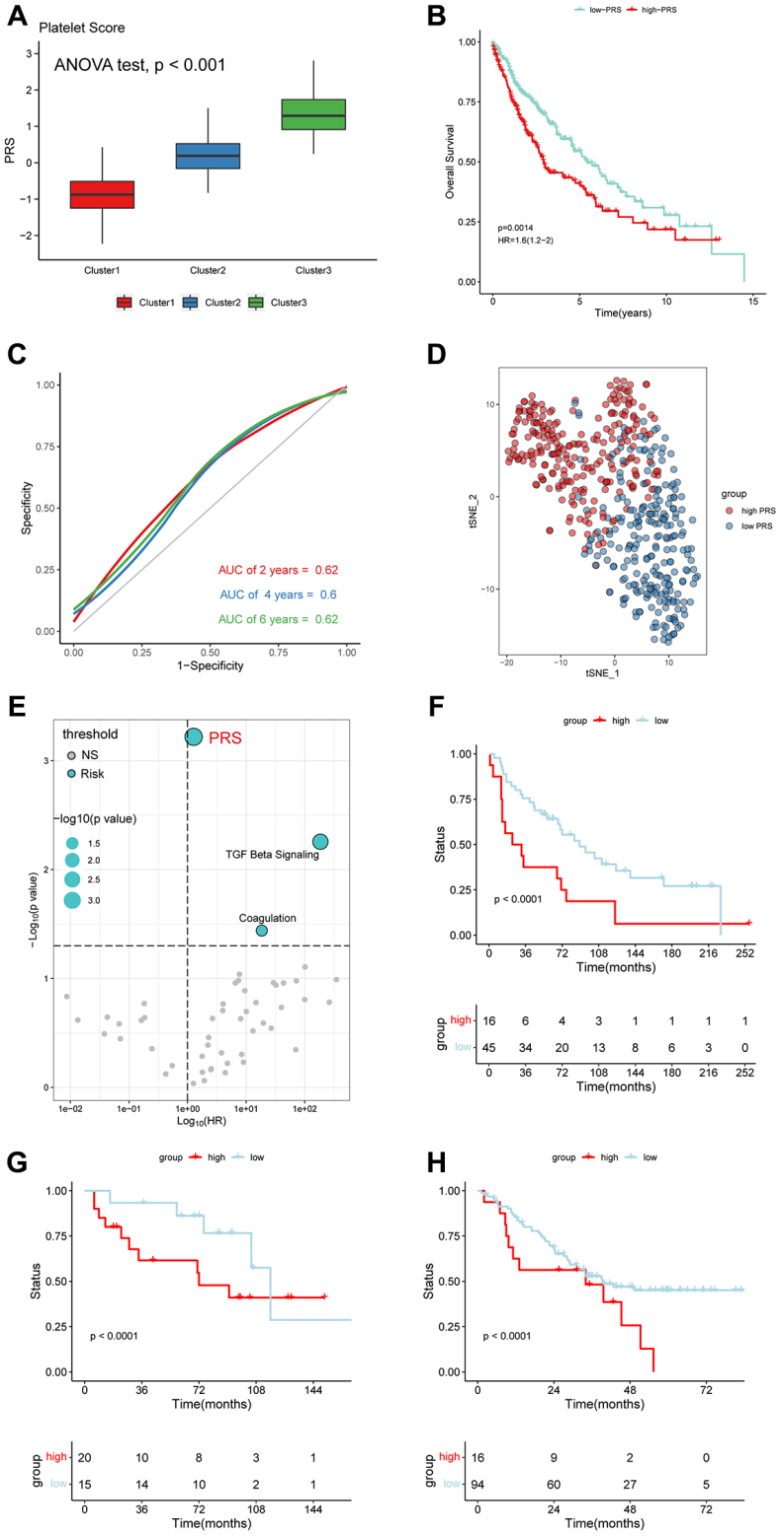
**Platelet-related scores (PRS) were an unfavorable factor in LUSC.** (**A**) The cluster 3 with worst outcomes had higher PRS. (ANOVA test, *p* < 0.001). (**B**) OS analysis for the LUSC patients with high- and low-PRS. (HR = 1.6). (**C**) The HR and *p*-value of PRS and 50 Hallmark pathways. (**D**) ROC curves for 2-, 4-, and 6-year. (**E**) PCA displayed the distribution of the two subclusters. (**F**–**H**) LUSC patients with high PRS tended to have a bad prognosis in GEO database (GSE3141, GSE12472 and GSE30219).

Based on the results from the previous section, we calculated platelet scores (PRS) for each LUSC patient using the ssGSEA algorithm. By comparing the PRS between the three clusters, we found that cluster 1 had the lowest scores, while cluster 3 had the highest scores (Figure 4A). Using the median of PRS, we divided LUSC patients into high PRS and low PRS subgroups. Survival analysis showed that patients with high PRS had worse clinical outcomes (*p* = 0.0014, HR = 1.6) (Figure 4B), and ROC curves suggested that PRS had moderate performance (Figure 4C). We also performed PCA algorithm to visualize the distribution of the two subgroups (Figure 4D). Using the univariate Cox method, we calculated the HR and *p*-values of PRS and 50 oncogenic pathways. Compared to 50 oncogenic pathways, PRS was the most significant prognostic risk factor (Figure 4E). The KM analysis based on three external datasets confirmed that PRS was significantly associated with unfavorable outcomes in LUSC (Figure 4F–4H).

### Relevance of the platelet scores and biological processes

**Figure 5:**
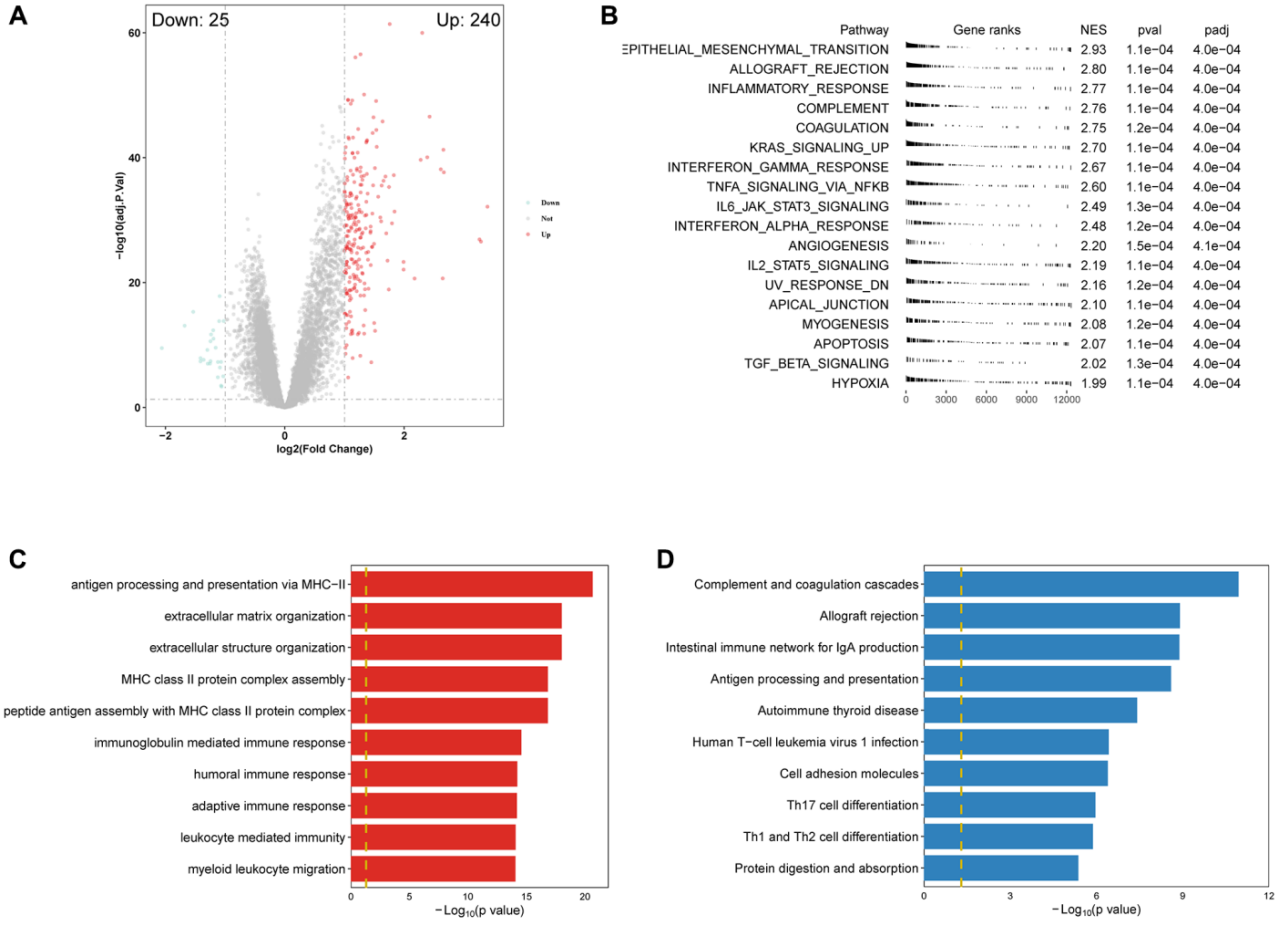
**Enrichment analysis revealed PRS was significantly correlated with immune-related pathways.** (**A**) Volcano plot of DEGS between high- and low-PRS patients. (**B**) GSEA results showed the significantly different Hallmark genesets (|NES| > 1 and adjusted *p* < 0.001). (**C**, **D**) The top 10 results of GO and KEGG enrichment based on PRS-related DEGs.

Using differential analysis, we identified 265 differentially expressed genes (DEGs), with the majority being upregulated (Figure 5A). We conducted GSEA algorithm to screen out significantly enriched cancer-related pathways based on the principle of |NES| > 1 and adjusted *p*-value < 0.001. All of the pathways were enriched in the high PRGs group (Figure 5B). We also employed GO and KEGG analyses to explore the association of PRS with biological function (Figure 5C, 5D). The top 3 GO results were antigen processing and presentation via MHC−II, extracellular matrix organization, and immunoglobulin mediated immune response, while the top 3 KEGG results were complement and coagulation cascades, allograft rejection, and intestinal immune network for IgA production. These results further demonstrated that PRS were involved in multiple biological processes, especially in the immune response.

### Distinct TME pattern related to platelet scores

**Figure 6:**
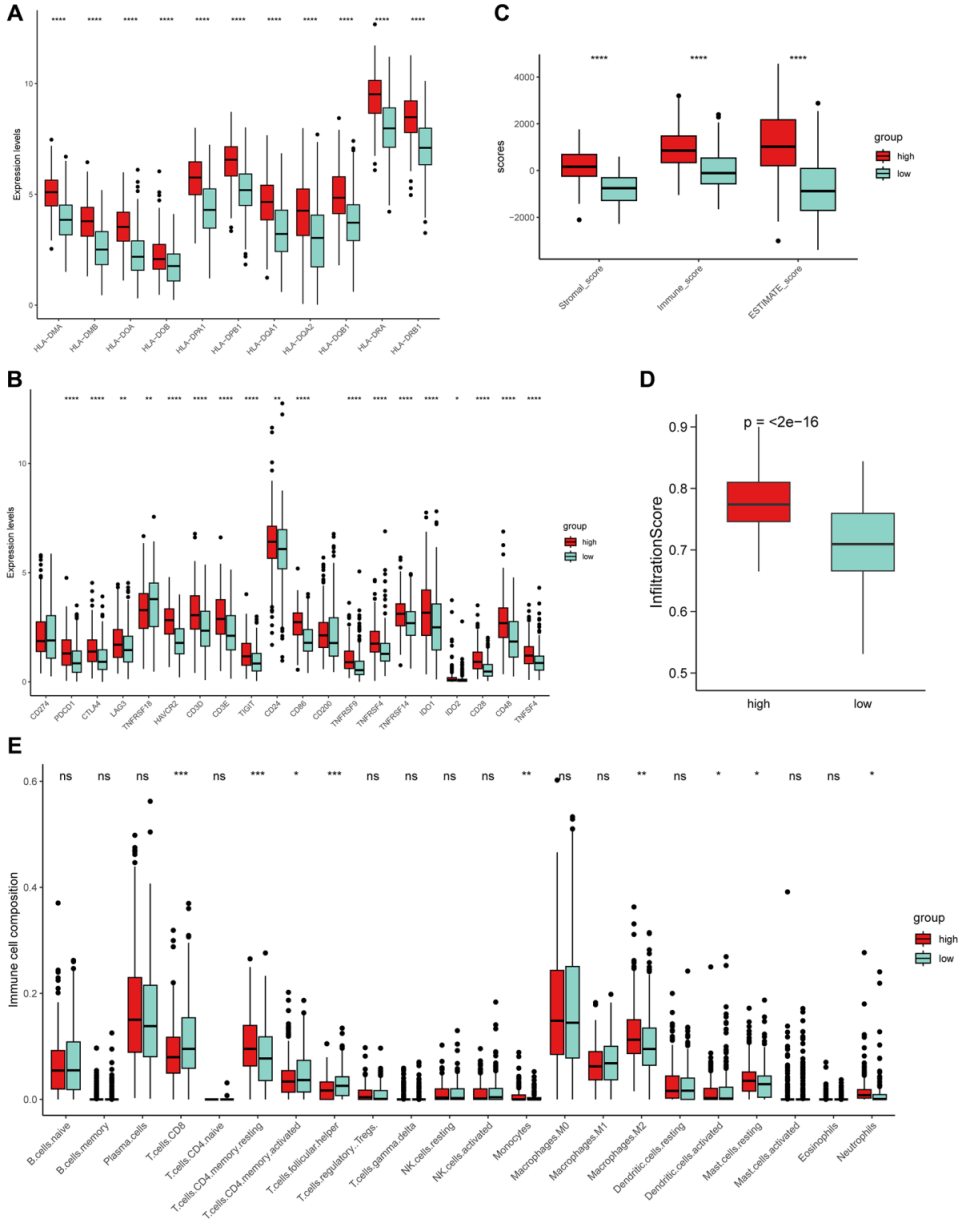
**The exploration of PRS and tumor microenvironment (TME).** (**A**) MHC-II molecules were highly expressed in high PRS patients. (**B**) Similarly, almost all immune checkpoints (ICs) were highly expressed in high PRS patients. Boxplot showed the high PRS patients had significantly higher infiltration levels of TME using ESTIMATE (**C**) and ImmuCellAI (**D**) algorithm. (**E**) Two types of PRS patients exhibited distinct immune cell populations. Wilcoxon test, ^*^P < 0.05, ^**^P < 0.01, ^***^P < 0.001, ^****^P < 0.0001 and ns represents not significant.

It is well known that the tumor microenvironment (TME) plays a crucial role in tumor progression and response to immunotherapy [40–42]. Given the potential significance of PRS in immunity, we further explored the relationship between PRS and TME. We compared the expression profiles of MHC II molecules in the two subtypes and found that high PRS had higher expression (Figure 6A). Similarly, high PRS generally had higher expression levels of immune checkpoint molecules (Figure 6B). We further implemented ESTIMATE method to calculate the Stromal score, Immune score and ESTIMATE score for LUSC patients, and found the high PRS had higher infiltration level than low PRS (Figure 6C). We demonstrated that high PRS were significantly positively correlated with infiltration scores through another algorithm named ImmuCellAI [43] (Figure 6D). Furthermore, we compared the infiltration levels of 22 immune cells in TME using the CIBERSORT algorithm. The result illustrated that the two PRS subgroups had distinct immune infiltration patterns, and the compositions of 9 types of immune cells were significantly different (Figure 6E).

### Construction of risk signature and evaluation of performance

**Figure 7:**
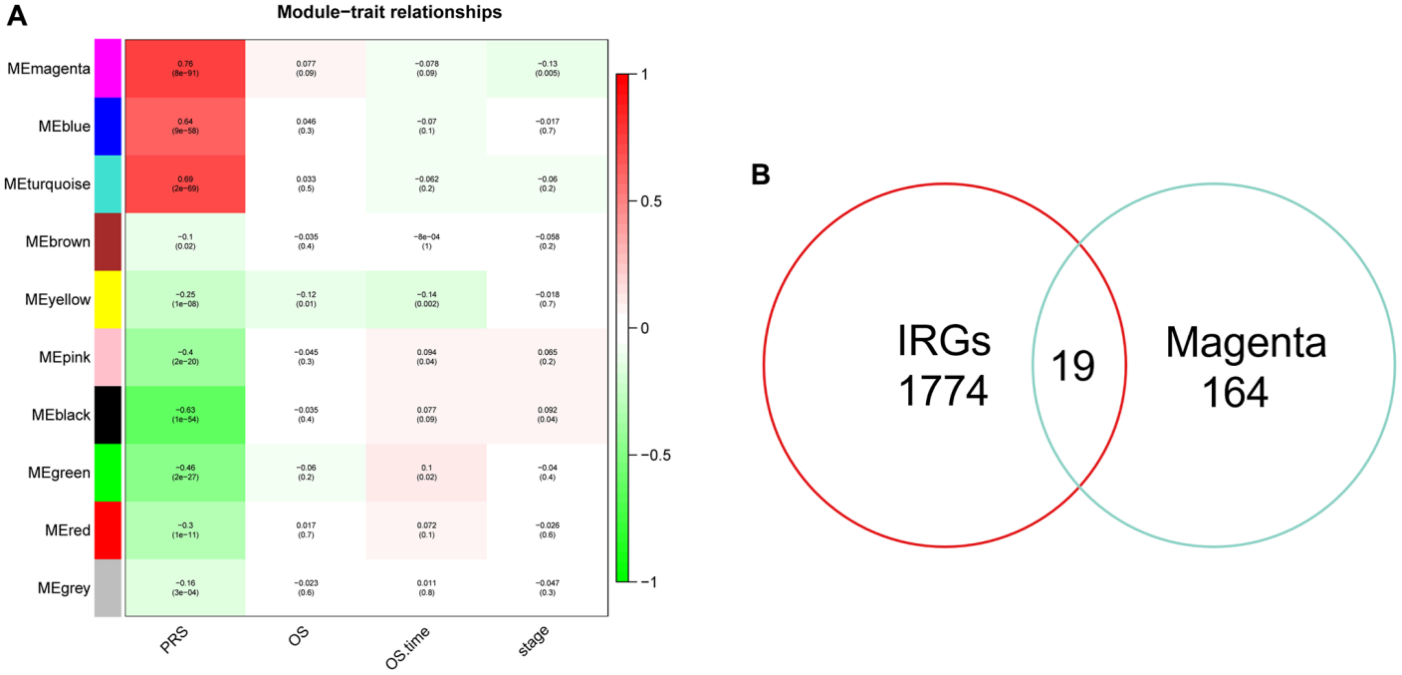
**Identification of hub genes related to PRS by WGCNA.** (**A**) Heatmap showed the correlation coefficient and *p*-value of distinct module. (**B**) Venn plots of 10 common genes related to PRS and immunity.

Given the important connection between PRS and immunity, we conducted the WGCNA algorithm to investigate genes related to platelets and immunity. The heatmap showed the correlation coefficient and *p*-value of each module feature with PRS, and the magenta module had highest correlation (Figure 7A). By intersecting the immune-related genes (IRGs) and genes in the magenta module, we got 19 genes in total (Figure 7B).

**Figure 8:**
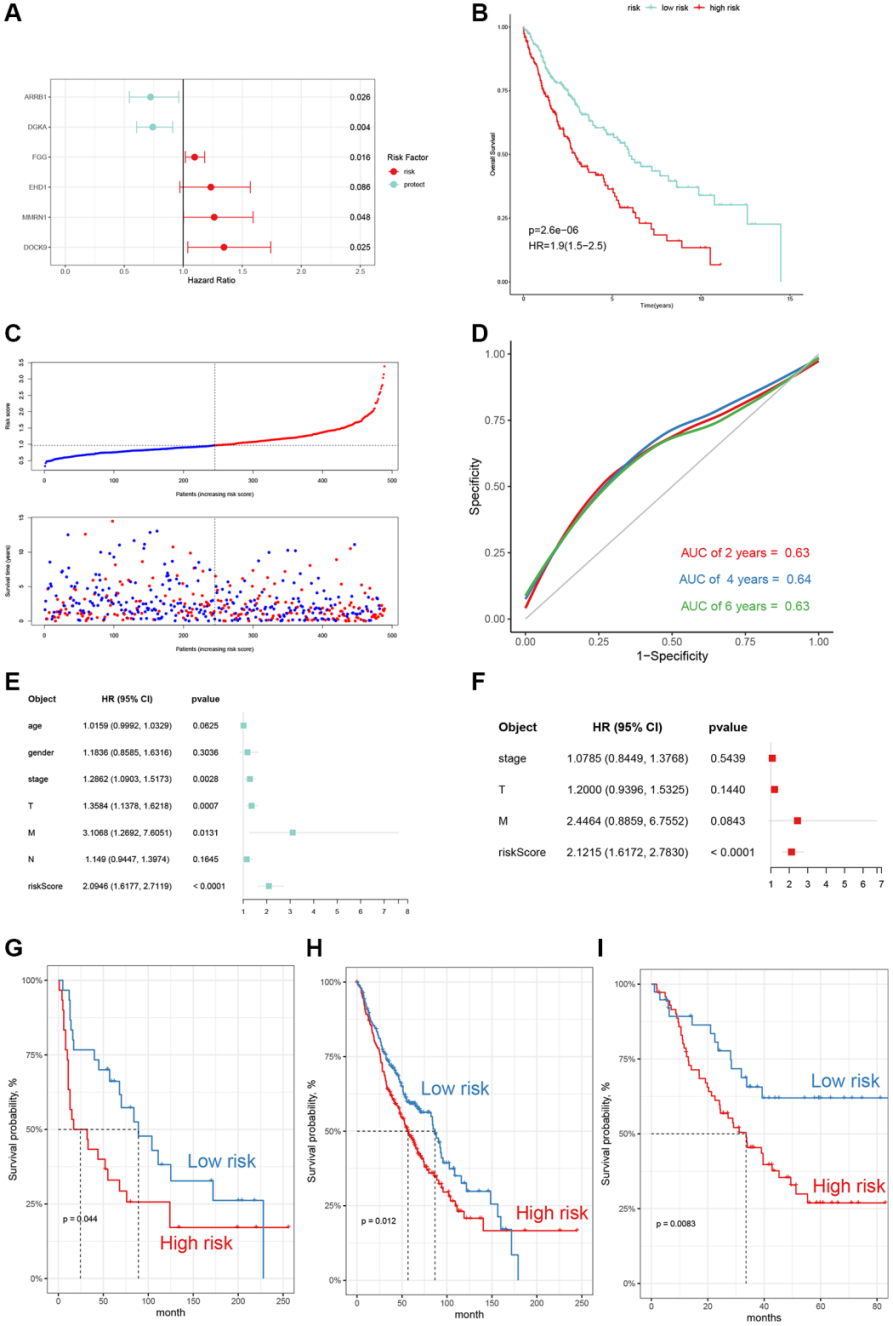
**Construction and evaluation of the 6-gene signature.** (**A**) The multivariate Cox risk model of 6 genes. (**B**) OS curves of the two risk subgroups (HR = 1.9, *p*-value < 0.001). (**C**) The distribution of risk scores, survival times and outcomes of LUSC patients. (**D**) Receiver operating characteristic (ROC) curves for 2-, 4- and 6-year of TCGA-LUSC cohort. (**E**, **F**) Univariate and multivariate Cox regression analyses of the risk model and other clinic-pathological factors. (**G**–**I**) External validation in GEO database (GSE30219, GSE157011, and GSE3141).

To better predict the clinical outcomes in LUSC patients, we constructed a 6-gene risk signature using the multivariate Cox regression model (Figure 8A). The risk score of each LUSC patient was calculated using the following specific formula: Risk scores = (−0.3242) × Exp ARRB1 + (−0.2985) × Exp DGKA + 0.0922 × Exp FGG + 0.2103 × Exp EHD1 + 0.2332 × Exp MMRN1 + 0.2964 × Exp DOCK9. We dichotomized the LUSC patients into high and low risk subgroups. OS analysis illustrated that risk score was associated with unfavorable outcomes (*p* < 0.001, HR = 1.9) (Figure 8B). The distribution of risk score and survival status of LUSC patients are shown in Figure 8C. ROC curves were employed to evaluate the performance of the 6-gene signature, and the AUC value of 2-, 4-, and 6-year was separately 0.63, 0.64, and 0.63 (Figure 8D). We also combined the univariate Cox and multivariate Cox analyses to demonstrated that the 6-gene signature was an independent risk factor in LUSC (Figure 8E, 8F). Survival analysis results on three external datasets demonstrated the robustness of our model (Figure 8G–8I).

**Figure 9:**
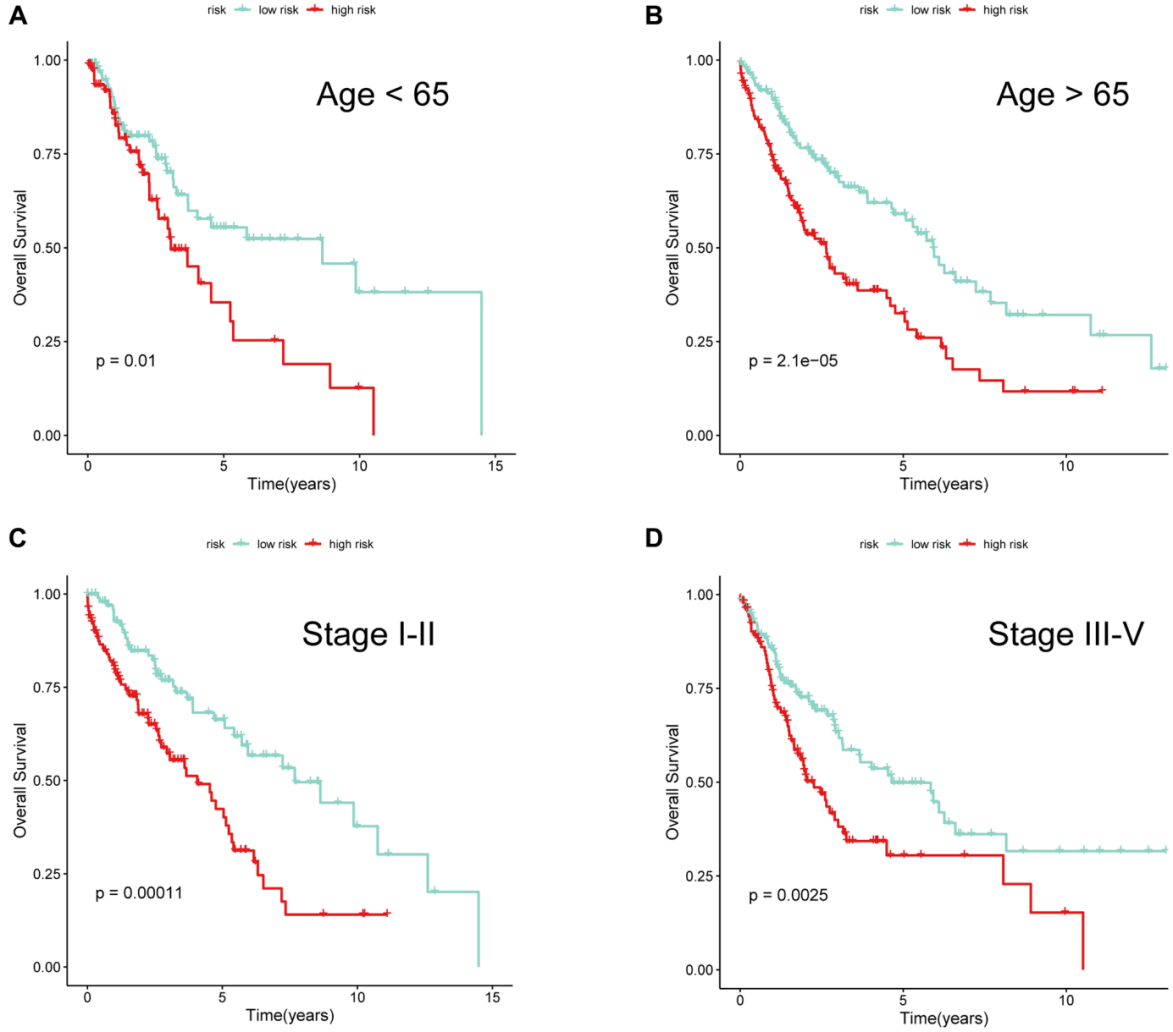
**The performance of 6-gene signature under different clinical characteristics.** KM survival curves for the two risk subgroups of LUSC patients stratified by age (**A**, **B**) and tumor stage (**C**, **D**).

To further investigate whether the risk signature was applicable for LUSC patients stratified by different clinical features, we performed survival analyses under various clinical subgroups, including gender (Female/Male) and stage (I–II/III–IV). In each stratum of the above clinical features, the high-risk subtype had significant worse clinical outcomes than the low-risk subtype (Figure 9A–9D). These results demonstrated that our risk signature had reliable predictive ability for prognosis within each stratum.

### Relationship of risk signature with TME and prediction of immunotherapy response

**Figure 10:**
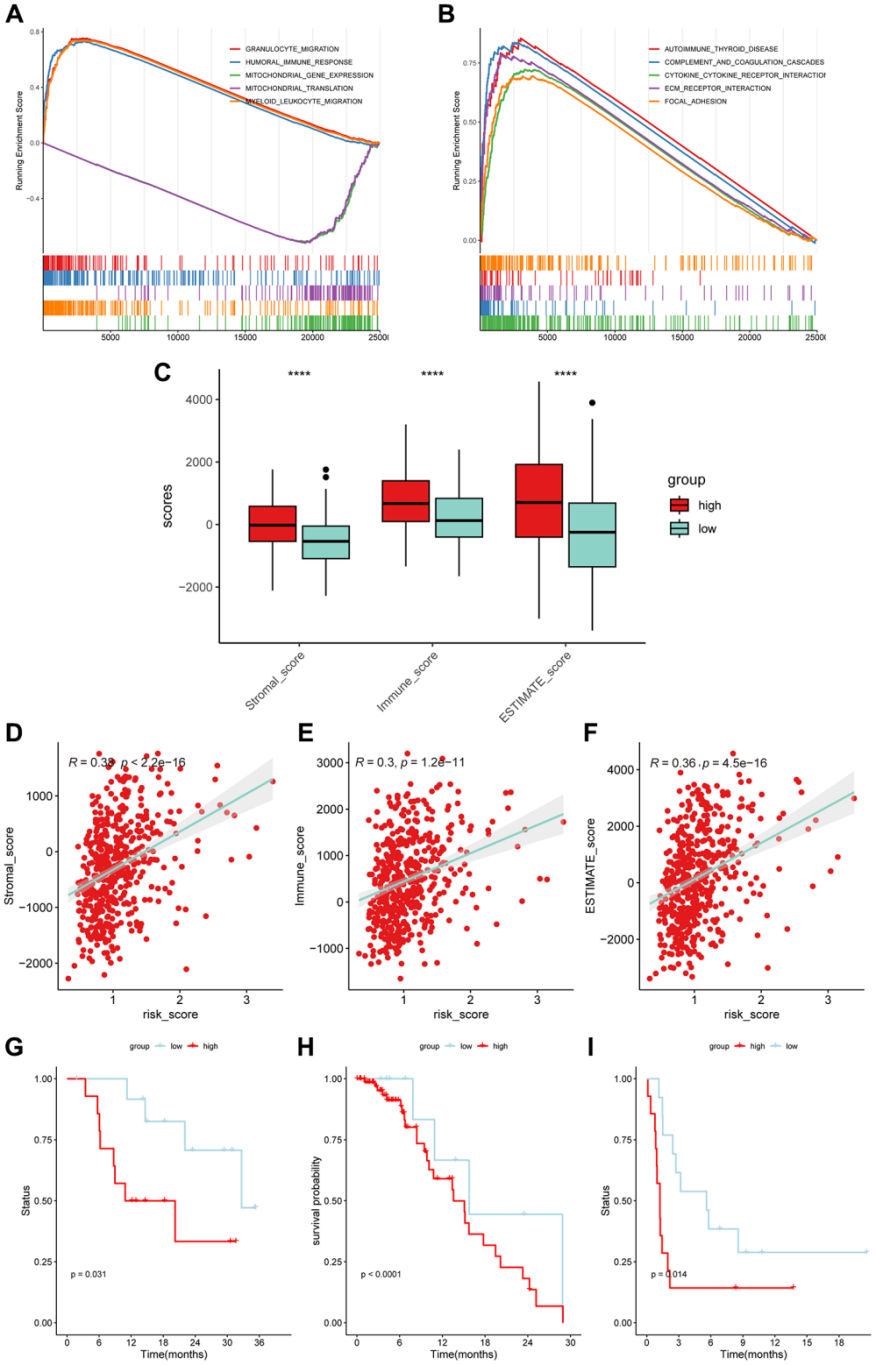
**The application of 6-gene signature in immunotherapy efficacy.** The top 5 results of GSEA analysis in the term of GO-BP (**A**) and KEGG (**B**). (**C**) The high-risk subgroups had higher Stromal scores, Immune scores, and ESTIMATE scores (Wilcoxon test). (**D**–**F**) The risk scores of LUSC patients were positively correlated with the infiltration scores. (**G**–**I**) The low-risk subgroups were more prominent in prognosis than the high-risk subgroups in immunotherapy cohorts (GSE78220: Melanoma; GSE176307: Urothelial Cancer; GSE135222: Non-small cell lung carcinoma).

We conducted GSEA analysis to investigate the difference of biological function in the term of GO and KEGG between the two risk subgroups (Figure 10A, 10B). The results showed significant differences in immune-related processes, such as granulocyte migration, humoral immune response, and autoimmune thyroid disease. We then compared the infiltration levels of the TME and found that the high-risk subgroup had significantly higher scores (Figure 10C). The scatter plot also illustrated that risk scores were positively correlated with TME scores using Spearman analysis (Figure 10D–10F). Next, we explored the performance of the 6-gene signature in predicting the response to immunotherapy. The KM analysis results showed a significant correlation between the gene signature and the efficacy of immunotherapy in LUSC patients (Figure 10G–10I).

### scRNA data analysis

**Figure 11:**
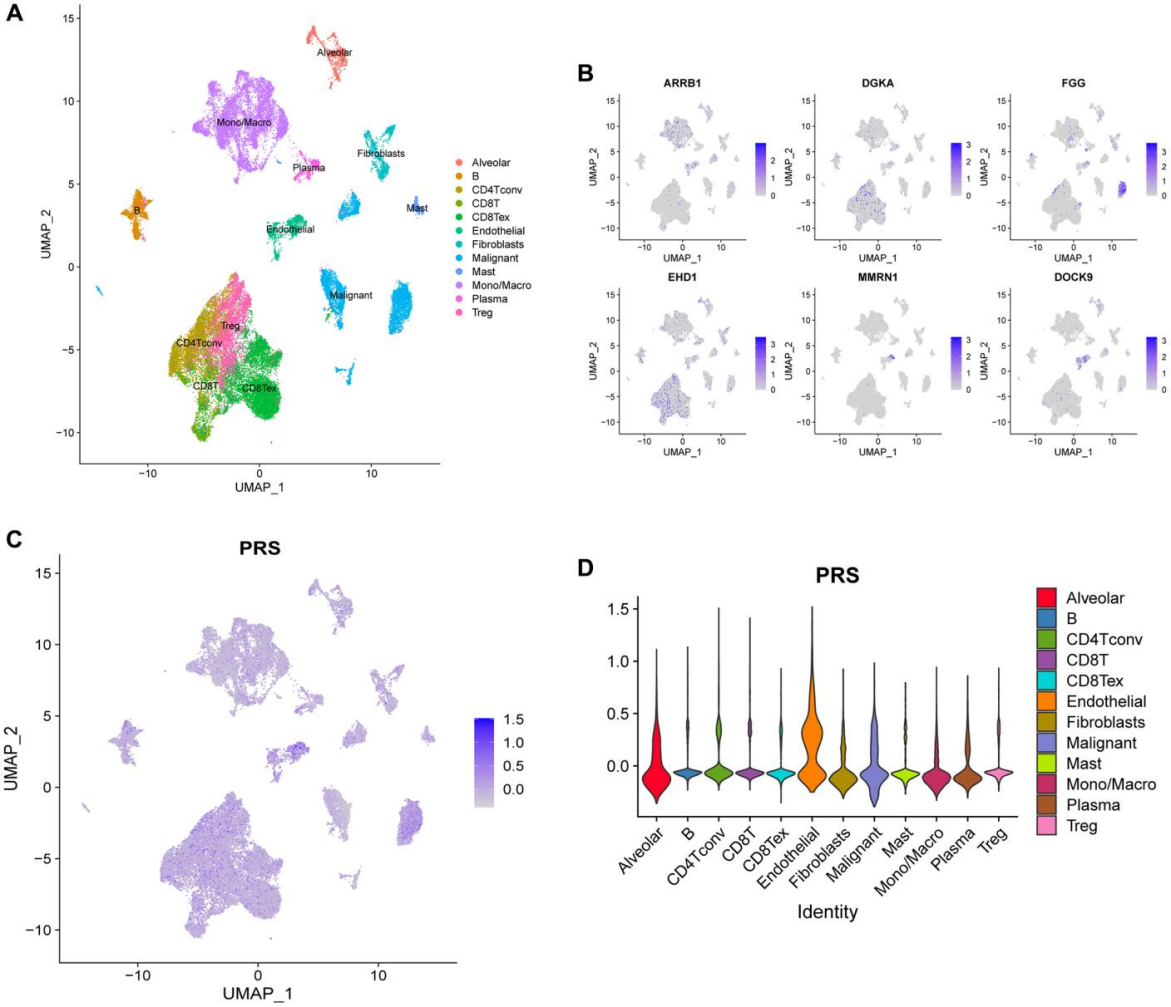
**scRNA analysis of PRS in E-MTAB-6149 dataset.** (**A**) A total of 12 cell types was confirmed. (**B**) The expression distributions of 6 genes across different cell types. (**C**, **D**) The platelet-related scores (PRS) calculated by AddModuleScore function in Seurat among 12 cell types.

We retrieved single-cell expression data from 5 NSCLC patients in the E-MTAB-6149 cohort, which included approximately 40,000 cells for subsequent analysis. Using the Seurat package, we confirmed the presence of 12 distinct cell types and visualized these cells using the UMAP algorithm (Figure 11A). We also examined the expression of 6 genes using the FeaturePlot function in Seurat, which showed that ARRB1 was highly expressed in monocyte/macrophage and endothelial cells, while FGG was mainly expressed in malignant cells (Figure 11B). To estimate the PRS in different cell types, we used the AddModuleScore procedure, which indicated that PRS were most abundant in endothelial, alveolar, and malignant cells (Figure 11C, 11D). Endothelial and alveolar cells were in contact with capillaries and therefore had a higher PRS score. Interestingly, malignant cells also showed high expression of PRGs, suggesting that tumors might affect the TME and promote progression by secreting PRGs.

### Drug sensitivity speculation

**Figure 12:**
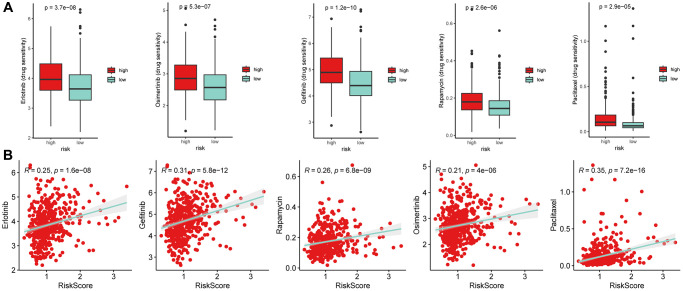
**Drug susceptibility analysis.** (**A**) The half maximal inhibitory concentration (IC50) values of several anti-tumor drugs (Erlotinib, Osimertinib, Gefitinib, Rapamycin, and Paclitaxel) (Wilcoxon test). (**B**) The Spearman correlation analysis of risk scores with these drugs. The results showed that high-risk subgroups were resistance to the five drugs.

We used pharmacogenomic data from GDSC2 (https://www.cancerrxgene.org/) to perform ridge regression analysis using the “oncoPredict” package to predict the susceptibility of anti-tumor drugs in LUSC patients. The results showed that the risk scores were significantly positively correlated with several anti-tumor drugs, including Erlotinib, Osimertinib, Gefitinib, Rapamycin, and Paclitaxel (Figure 12A, 12B). These findings suggest that PRGs may be potential therapeutic targets in LUSC and may help doctors to better personalize chemotherapy strategies for individual patients.

## DISCUSSION

The results of our study have provided important insights into the role of platelet-related genes in the prognosis and response to immunotherapy in patients with LUSC. Our comprehensive analysis of public gene expression datasets and bioinformatics tools has identified a total of 19 genes related to platelet and immunity that were significantly associated with LUSC prognosis. These genes were used to establish a prognostic gene signature that was validated using multiple independent datasets and had good performance in both training and external datasets.

One of the major challenges in improving the prognosis of LUSC patients is the lack of reliable biomarkers for predicting the progression of the disease [44, 45]. Accurate prognostic biomarkers could assist in the selection of the most appropriate treatment for each individual patient, monitor disease progression and response to treatment, and ultimately improve patient outcomes [46]. Recent studies have developed risk model based on PRGs in multiple cancer [22, 23, 47, 48], indicating platelet-related genes may have prognostic value in LUSC. Clinically, it has been found that a high platelet count is associated with poor prognosis or metastasis across a multitude of cancer types, including lung, colorectal, breast, pancreatic, and kidney cancers [49–55]. Platelets are known to play a significant role in influencing both the disease burden and the efficacy of treatments for cancer patients [56]. However, the prognostic implications of PRGs in LUSC and their underlying mechanisms remain under-investigated and poorly understood.

We identified 122 differentially expressed PRGs, indicating widespread dysregulation of PRGs in LUSC patients. The result was consistent with previous reports of dysregulated PRGs in other types of cancer. [23, 28, 48]. The Cox analysis results showed that most PRGs were associated with unfavorable outcomes in LUSC, consistent with reports in LUAD [28]. Cancer cells can induce platelet formation by secreting related cytokines, which contribute to metastasis [57]. In addition, excessive activation of platelets, which can lead to blood clots, is a leading cause of death in patients receiving chemotherapy [10]. Targeting these PRGs might play an important role in suppressing tumor metastasis and decreasing the side effects of treatments. Combined with the high PRS being associated with poor prognosis, high PRS patients might be suitable to accept treatment with antiplatelet drugs such as aspirin and clopidogrel. As mentioned earlier, platelets play an important role in tumor immunity. The results of enrichment analysis demonstrate that PRGs and PRS are involved in immune-related pathways, such as antigen processing and presentation via MHC-II, as well as complement and coagulation cascades. Antigen processing and presentation via MHC-II help in identifying tumor cells and enhancing anti-tumor immunity [58]. Previous bioinformatic analysis studies have also shown that the complement and coagulation cascades pathway is one of the key pathways in lung cancer [59]. Our findings deepen the understanding of the relationship between PRGs and immunity. In the future, it will be interesting and important to conduct further research on these PRGs.

We divided LUSC patients into three clusters based on PRGs, and the three clusters showed significantly in prognosis and biological function. Moreover, cluster 3, with the worst prognosis, had the highest PRS. For example, cluster 3 had high enrichment scores of the JAK-STAT signaling pathway, which was dangerous and carcinogenic in lung cancer [60, 61]. Cell adhesion molecules cams were also enriched in cluster 3, consistent with the role of platelets in promoting the invasion and migration of tumor cells. For example, Myriam Labelle et al. reported that platelets could intersect with malignant and promote the EMT [11]. Interestingly, we found that immune-related pathways were also correlated with PRGs, such as intestinal immune network for IgA production and leukocyte transendothelial migration. There are emerging studies that platelets can directly interact with immune cells or release cytokines to regulate immunity [62]. Our analysis provides a basis for further investigation of the role of PRGs in the immune response of LUSC patients.

According to the results of WGCNA, we obtained 19 genes related to platelet and immunity. By conducting Cox regression model, we established a 6-gene signature for predicting the prognosis and immunotherapy efficacy in LUSC. By performing multiple bioinformatics tools in training and external datasets, we confirmed that the risk signature was robust and reliable in LUSC patients. The six genes were ARRB1, DGKA, FGG, EHD1, MMRN1, and DOCK9. Among them, ARRB1 and DGKA are prognostic protective factors, while the remaining four genes are risk factors. ARRB1 encodes a member of the beta-arrestin family of proteins, which are known to regulate various signaling pathways involved in cell growth, survival, and death [63]. Previous study indicated that loss of beta-arrestin1 expression was associated with poorer prognosis for both LUAD and LUSC [64]. In addition, ARRB1 was a potential biomarker in lung cancer to distinguish LUAD from LUSC [65]. DGKA (Diacylglycerol Kinase Alpha) is a gene that encodes an enzyme that is involved in regulating the signaling pathways of diacylglycerol. Jingping Yun et al. found that DGKA was associated with unfavorable clinical outcomes and promoted metastasis in NSCLC [66]. It is worth noting that the expression data and clinical information used by the authors are obtained from in-house samples. In addition, our results were calculated based on multivariate Cox analysis, in which the linear correlations between genes affect them. Interestingly, DGKA had a dual function during cisplatin resistance in two lung cancer cell lines [67]. So, more clinical samples and experiments are needed to address the function and role of DGKA in LUSC. Fibrinogen gamma chain (FGG) is a component of the fibrinogen protein, which is involved in blood clotting. Recent studies have indicated a potential role for FGG in the development and progression of malignant, especially in EMT [68, 69]. Fibrinogen also promote tumor growth and metastasis in lung cancer [70]. Two studies have identified FGG as useful biomarker for early diagnosis and prognosis of the disease [71, 72]. Ying Xing et al. found that the EHD1 expression level in metastatic patients was higher than that of non-metastatic patients in lung cancer [73]. Targeting EHD1 was considered a remedy for advanced metastatic patients. Additionally, EHD1 can promote angiogenesis and resistance to chemotherapy [74, 75]. MMRN1 (Multimerin 1) is a massive, soluble protein to promote platelet adhesion. MMRN1 was upregulated in ovarian cancer [76] and was an unfavorable factor in AML [77]. Several studied suggested that MMRN1 might be potential diagnostic and prognostic biomarker in multiple cancer [78, 79]. In lung cancer, Andres Metspalu et al. found that combining a panel of dysregulated genes, including MMRN1, predicted prognosis better than histological stage [80]. DOCK9 (Dedicator of cytokinesis 9) is a gene that encodes a guanine nucleotide exchange factor (GEF) protein that is involved in the regulation of actin cytoskeleton dynamics. GEFs was dysregulated in human cancer and contribute to tumor invasion and metastasis [81]. DOCK9 is a risk factor in high-grade soft-tissue sarcoma [82]. Recently research found that DOCK9 was upregulated in LUSC compare with normal tissues [83]. The function and mechanism of DOCK9 in lung cancer, remain more experiments to investigate. Given these, the 6-gene signature involved multiple cancer-related processes and was closely related to prognosis in lung cancer.

There was a complex cell interaction or communication between tumor cells and the tumor microenvironment (TME), which had a huge influence on tumor invasion and progression, as well as on response to immunotherapy [40]. We found that high PRS patients had higher immune infiltration scores but more tumor-promoting cells (such as M2 macrophages and neutrophils), while low PRS patients had more cytotoxic cells (such as CD8+ T cells and follicular helper CD4+ T cells). Recent studies revealed that neutrophils facilitated lymphatic metastasis and were a risk factor in bladder cancer [84, 85]. This could contribute to the differences in prognosis among LSUC patients. Additionally, our 6-gene signature was significantly correlated with TME and was able to predict immunotherapy efficacy in LUSC. scRNA data analysis further suggested that tumor cells might affect TME by expressing PRGs. A comprehensive examination of the underlying mechanisms of TME is expected to enhance immunotherapy approaches, ultimately leading to prolonged positive outcomes for LUSC patients [86].

However, there are some limitations to this study that need to be acknowledged. Firstly, the gene signature established in this study needs to be validated in larger and more diverse patient populations to fully establish its clinical utility. Additionally, further research is needed to understand the biological mechanisms underlying the association between platelet-related genes and LSCC prognosis. Finally, further experiments are needed to fully understand the mechanism of the 6-gene signature in immune response in LUSC patients. In future work, we will collect samples from LUSC patients to validate our prognostic signature and conduct experiments to explore the connection between the function of PRGs genes and immunity.

In conclusion, our study highlights the significance of PRGs in LUSC prognosis and suggests that PRS could serve as a valuable prognostic risk factor for personalized treatment in LUSC patients. The risk signature developed in this study has the potential to be used as a tool for risk assessment and personalized treatment. However, further validation is necessary to confirm its utility in clinical settings. Our findings emphasize the need for further investigation into the mechanisms underlying the relationships between PRS, immune response, and LUSC prognosis.

## Supplementary Materials

Supplementary Figure 1

Supplementary Table 1

## References

[1] Sung H, Ferlay J, Siegel RL, Laversanne M, Soerjomataram I, Jemal A, Bray F. Global Cancer Statistics 2020: GLOBOCAN Estimates of Incidence and Mortality Worldwide for 36 Cancers in 185 Countries. CA Cancer J Clin. 2021; 71:209–49. 10.3322/caac.21660 33538338

[2] Conti L, Gatt S. Squamous-Cell Carcinoma of the Lung. N Engl J Med. 2018; 379:e17. 10.1056/NEJMicm1802514 30207918

[3] Mao Y, Yang D, He J, Krasna MJ. Epidemiology of Lung Cancer. Surg Oncol Clin N Am. 2016; 25:439–45. 10.1016/j.soc.2016.02.001 27261907

[4] Reck M, Remon J, Hellmann MD. First-Line Immunotherapy for Non-Small-Cell Lung Cancer. J Clin Oncol. 2022; 40:586–97. 10.1200/JCO.21.01497 34985920

[5] Yang S, Zhang Z, Wang Q. Emerging therapies for small cell lung cancer. J Hematol Oncol. 2019; 12:47. 10.1186/s13045-019-0736-3 31046803 PMC6498593

[6] Suresh K, Naidoo J, Lin CT, Danoff S. Immune Checkpoint Immunotherapy for Non-Small Cell Lung Cancer: Benefits and Pulmonary Toxicities. Chest. 2018; 154:1416–23. 10.1016/j.chest.2018.08.1048 30189190 PMC6335259

[7] Smyth SS, McEver RP, Weyrich AS, Morrell CN, Hoffman MR, Arepally GM, French PA, Dauerman HL, Becker RC, and 2009 Platelet Colloquium Participants. Platelet functions beyond hemostasis. J Thromb Haemost. 2009; 7:1759–66. 10.1111/j.1538-7836.2009.03586.x 19691483

[8] Semple JW, Italiano JE Jr, Freedman J. Platelets and the immune continuum. Nat Rev Immunol. 2011; 11:264–74. 10.1038/nri2956 21436837

[9] Best MG, Wesseling P, Wurdinger T. Tumor-Educated Platelets as a Noninvasive Biomarker Source for Cancer Detection and Progression Monitoring. Cancer Res. 2018; 78:3407–12. 10.1158/0008-5472.CAN-18-0887 29921699

[10] Suzuki-Inoue K. Platelets and cancer-associated thrombosis: focusing on the platelet activation receptor CLEC-2 and podoplanin. Blood. 2019; 134:1912–8. 10.1182/blood.2019001388 31778548

[11] Labelle M, Begum S, Hynes RO. Direct signaling between platelets and cancer cells induces an epithelial-mesenchymal-like transition and promotes metastasis. Cancer Cell. 2011; 20:576–90. 10.1016/j.ccr.2011.09.009 22094253 PMC3487108

[12] Stone RL, Nick AM, McNeish IA, Balkwill F, Han HD, Bottsford-Miller J, Rupairmoole R, Armaiz-Pena GN, Pecot CV, Coward J, Deavers MT, Vasquez HG, Urbauer D, et al. Paraneoplastic thrombocytosis in ovarian cancer. N Engl J Med. 2012; 366:610–8. 10.1056/NEJMoa1110352 22335738 PMC3296780

[13] Tesfamariam B. Involvement of platelets in tumor cell metastasis. Pharmacol Ther. 2016; 157:112–9. 10.1016/j.pharmthera.2015.11.005 26615781

[14] Hinterleitner C, Strähle J, Malenke E, Hinterleitner M, Henning M, Seehawer M, Bilich T, Heitmann J, Lutz M, Mattern S, Scheuermann S, Horger M, Maurer S, et al. Platelet PD-L1 reflects collective intratumoral PD-L1 expression and predicts immunotherapy response in non-small cell lung cancer. Nat Commun. 2021; 12:7005. 10.1038/s41467-021-27303-7 34853305 PMC8636618

[15] Schmied L, Höglund P, Meinke S. Platelet-Mediated Protection of Cancer Cells From Immune Surveillance - Possible Implications for Cancer Immunotherapy. Front Immunol. 2021; 12:640578. 10.3389/fimmu.2021.640578 33777033 PMC7988080

[16] Bahmani B, Gong H, Luk BT, Haushalter KJ, DeTeresa E, Previti M, Zhou J, Gao W, Bui JD, Zhang L, Fang RH, Zhang J. Intratumoral immunotherapy using platelet-cloaked nanoparticles enhances antitumor immunity in solid tumors. Nat Commun. 2021; 12:1999. 10.1038/s41467-021-22311-z 33790276 PMC8012593

[17] Hu Q, Li H, Archibong E, Chen Q, Ruan H, Ahn S, Dukhovlinova E, Kang Y, Wen D, Dotti G, Gu Z. Inhibition of post-surgery tumour recurrence via a hydrogel releasing CAR-T cells and anti-PDL1-conjugated platelets. Nat Biomed Eng. 2021; 5:1038–47. 10.1038/s41551-021-00712-1 33903744 PMC9102991

[18] Diem S, Schmid S, Krapf M, Flatz L, Born D, Jochum W, Templeton AJ, Früh M. Neutrophil-to-Lymphocyte ratio (NLR) and Platelet-to-Lymphocyte ratio (PLR) as prognostic markers in patients with non-small cell lung cancer (NSCLC) treated with nivolumab. Lung Cancer. 2017; 111:176–81. 10.1016/j.lungcan.2017.07.024 28838390

[19] Liu J, Li S, Zhang S, Liu Y, Ma L, Zhu J, Xin Y, Wang Y, Yang C, Cheng Y. Systemic immune-inflammation index, neutrophil-to-lymphocyte ratio, platelet-to-lymphocyte ratio can predict clinical outcomes in patients with metastatic non-small-cell lung cancer treated with nivolumab. J Clin Lab Anal. 2019; 33:e22964. 10.1002/jcla.22964 31282096 PMC6805305

[20] Cui MM, Li N, Liu X, Yun ZY, Niu Y, Zhang Y, Gao B, Liu T, Wang RT. Platelet distribution width correlates with prognosis of non-small cell lung cancer. Sci Rep. 2017; 7:3456. 10.1038/s41598-017-03772-z 28615714 PMC5471191

[21] Li W, Liu JB, Hou LK, Yu F, Zhang J, Wu W, Tang XM, Sun F, Lu HM, Deng J, Bai J, Li J, Wu CY, et al. Liquid biopsy in lung cancer: significance in diagnostics, prediction, and treatment monitoring. Mol Cancer. 2022; 21:25. 10.1186/s12943-022-01505-z 35057806 PMC8772097

[22] Du QC, Wang XY, Hu CK, Zhou L, Fu Z, Liu S, Wang J, Ma YY, Liu MY, Yu H. Integrative analysis of platelet-related genes for the prognosis of esophageal cancer. World J Clin Cases. 2022; 10:12077–88. 10.12998/wjcc.v10.i33.12077 36483802 PMC9724514

[23] Li X, Zhao K, Lu Y, Wang J, Yao W. Genetic Analysis of Platelet-Related Genes in Hepatocellular Carcinoma Reveals a Novel Prognostic Signature and Determines PRKCD as the Potential Molecular Bridge. Biol Proced Online. 2022; 24:22. 10.1186/s12575-022-00185-9 36463115 PMC9719151

[24] Wang P, Zhao W, Cao H. Development of a Platelet-Related Prognostic Model for Colorectal Cancer. Front Genet. 2022; 13:904168. 10.3389/fgene.2022.904168 35719389 PMC9198283

[25] Mayakonda A, Lin DC, Assenov Y, Plass C, Koeffler HP. Maftools: efficient and comprehensive analysis of somatic variants in cancer. Genome Res. 2018; 28:1747–56. 10.1101/gr.239244.118 30341162 PMC6211645

[26] Chen H, Yao J, Bao R, Dong Y, Zhang T, Du Y, Wang G, Ni D, Xun Z, Niu X, Ye Y, Li HB. Cross-talk of four types of RNA modification writers defines tumor microenvironment and pharmacogenomic landscape in colorectal cancer. Mol Cancer. 2021; 20:29. 10.1186/s12943-021-01322-w 33557837 PMC7869236

[27] Zhang G. Platelet-Related Molecular Subtype to Predict Prognosis in Hepatocellular Carcinoma. J Hepatocell Carcinoma. 2022; 9:423–36. 10.2147/JHC.S363200 35615530 PMC9126232

[28] Zhou C, Wang Y, Wang Y, Lei L, Ji MH, Zhou G, Xia H, Yang JJ. Predicting lung adenocarcinoma prognosis with a novel risk scoring based on platelet-related gene expression. Aging (Albany NY). 2021; 13:8706–19. 10.18632/aging.202682 33619234 PMC8034940

[29] Ritchie ME, Phipson B, Wu D, Hu Y, Law CW, Shi W, Smyth GK. limma powers differential expression analyses for RNA-sequencing and microarray studies. Nucleic Acids Res. 2015; 43:e47. 10.1093/nar/gkv007 25605792 PMC4402510

[30] Wilkerson MD, Hayes DN. ConsensusClusterPlus: a class discovery tool with confidence assessments and item tracking. Bioinformatics. 2010; 26:1572–3. 10.1093/bioinformatics/btq170 20427518 PMC2881355

[31] Hänzelmann S, Castelo R, Guinney J. GSVA: gene set variation analysis for microarray and RNA-seq data. BMC Bioinformatics. 2013; 14:7. 10.1186/1471-2105-14-7 23323831 PMC3618321

[32] Yoshihara K, Shahmoradgoli M, Martínez E, Vegesna R, Kim H, Torres-Garcia W, Treviño V, Shen H, Laird PW, Levine DA, Carter SL, Getz G, Stemke-Hale K, et al. Inferring tumour purity and stromal and immune cell admixture from expression data. Nat Commun. 2013; 4:2612. 10.1038/ncomms3612 24113773 PMC3826632

[33] Newman AM, Liu CL, Green MR, Gentles AJ, Feng W, Xu Y, Hoang CD, Diehn M, Alizadeh AA. Robust enumeration of cell subsets from tissue expression profiles. Nat Methods. 2015; 12:453–7. 10.1038/nmeth.3337 25822800 PMC4739640

[34] Yang Q, Gong H, Liu J, Ye M, Zou W, Li H. A 13-gene signature to predict the prognosis and immunotherapy responses of lung squamous cell carcinoma. Sci Rep. 2022; 12:13646. 10.1038/s41598-022-17735-6 35953696 PMC9372044

[35] Wang N, Zhu L, Wang L, Shen Z, Huang X. Identification of SHCBP1 as a potential biomarker involving diagnosis, prognosis, and tumor immune microenvironment across multiple cancers. Comput Struct Biotechnol J. 2022; 20:3106–19. 10.1016/j.csbj.2022.06.039 35782736 PMC9233189

[36] Fan T, Lu Z, Liu Y, Wang L, Tian H, Zheng Y, Zheng B, Xue L, Tan F, Xue Q, Gao S, Li C, He J. A Novel Immune-Related Seventeen-Gene Signature for Predicting Early Stage Lung Squamous Cell Carcinoma Prognosis. Front Immunol. 2021; 12:665407. 10.3389/fimmu.2021.665407 34177903 PMC8226174

[37] Langfelder P, Horvath S. WGCNA: an R package for weighted correlation network analysis. BMC Bioinformatics. 2008; 9:559. 10.1186/1471-2105-9-559 19114008 PMC2631488

[38] Satija R, Farrell JA, Gennert D, Schier AF, Regev A. Spatial reconstruction of single-cell gene expression data. Nat Biotechnol. 2015; 33:495–502. 10.1038/nbt.3192 25867923 PMC4430369

[39] Wu F, Fan J, He Y, Xiong A, Yu J, Li Y, Zhang Y, Zhao W, Zhou F, Li W, Zhang J, Zhang X, Qiao M, et al. Single-cell profiling of tumor heterogeneity and the microenvironment in advanced non-small cell lung cancer. Nat Commun. 2021; 12:2540. 10.1038/s41467-021-22801-0 33953163 PMC8100173

[40] Hinshaw DC, Shevde LA. The Tumor Microenvironment Innately Modulates Cancer Progression. Cancer Res. 2019; 79:4557–66. 10.1158/0008-5472.CAN-18-3962 31350295 PMC6744958

[41] Bader JE, Voss K, Rathmell JC. Targeting Metabolism to Improve the Tumor Microenvironment for Cancer Immunotherapy. Mol Cell. 2020; 78:1019–33. 10.1016/j.molcel.2020.05.034 32559423 PMC7339967

[42] Genova C, Dellepiane C, Carrega P, Sommariva S, Ferlazzo G, Pronzato P, Gangemi R, Filaci G, Coco S, Croce M. Therapeutic Implications of Tumor Microenvironment in Lung Cancer: Focus on Immune Checkpoint Blockade. Front Immunol. 2021; 12:799455. 10.3389/fimmu.2021.799455 35069581 PMC8777268

[43] Miao YR, Zhang Q, Lei Q, Luo M, Xie GY, Wang H, Guo AY. ImmuCellAI: A Unique Method for Comprehensive T-Cell Subsets Abundance Prediction and its Application in Cancer Immunotherapy. Adv Sci (Weinh). 2020; 7:1902880. 10.1002/advs.201902880 32274301 PMC7141005

[44] Socinski MA, Obasaju C, Gandara D, Hirsch FR, Bonomi P, Bunn PA Jr, Kim ES, Langer CJ, Natale RB, Novello S, Paz-Ares L, Pérol M, Reck M, et al. Current and Emergent Therapy Options for Advanced Squamous Cell Lung Cancer. J Thorac Oncol. 2018; 13:165–83. 10.1016/j.jtho.2017.11.111 29175116

[45] Lau SCM, Pan Y, Velcheti V, Wong KK. Squamous cell lung cancer: Current landscape and future therapeutic options. Cancer Cell. 2022; 40:1279–93. 10.1016/j.ccell.2022.09.018 36270277

[46] Niu Z, Jin R, Zhang Y, Li H. Signaling pathways and targeted therapies in lung squamous cell carcinoma: mechanisms and clinical trials. Signal Transduct Target Ther. 2022; 7:353. 10.1038/s41392-022-01200-x 36198685 PMC9535022

[47] Shu Y, Peng J, Feng Z, Hu K, Li T, Zhu P, Cheng T, Hao L. Osteosarcoma subtypes based on platelet-related genes and tumor microenvironment characteristics. Front Oncol. 2022; 12:941724. 10.3389/fonc.2022.941724 36212395 PMC9539847

[48] Xie J, Zou Y, Ye F, Zhao W, Xie X, Ou X, Xie X, Wei W. A Novel Platelet-Related Gene Signature for Predicting the Prognosis of Triple-Negative Breast Cancer. Front Cell Dev Biol. 2021; 9:795600. 10.3389/fcell.2021.795600 35096824 PMC8790231

[49] Yuan Y, Zhong H, Ye L, Li Q, Fang S, Gu W, Qian Y. Prognostic value of pretreatment platelet counts in lung cancer: a systematic review and meta-analysis. BMC Pulm Med. 2020; 20:96. 10.1186/s12890-020-1139-5 32312252 PMC7171794

[50] Belluco C, Forlin M, Delrio P, Rega D, Degiuli M, Sofia S, Olivieri M, Pucciarelli S, Zuin M, De Manzoni G, Di Leo A, Scabini S, Zorcolo L, Restivo A. Elevated platelet count is a negative predictive and prognostic marker in locally advanced rectal cancer undergoing neoadjuvant chemoradiation: a retrospective multi-institutional study on 965 patients. BMC Cancer. 2018; 18:1094. 10.1186/s12885-018-5022-1 30419864 PMC6233528

[51] Liu S, Fang J, Jiao D, Liu Z. Elevated Platelet Count Predicts Poor Prognosis in Breast Cancer Patients with Supraclavicular Lymph Node Metastasis. Cancer Manag Res. 2020; 12:6069–75. 10.2147/CMAR.S257727 32765104 PMC7381764

[52] Chen S, Na N, Jian Z. Pretreatment platelet count as a prognostic factor in patients with pancreatic cancer: a systematic review and meta-analysis. Onco Targets Ther. 2018; 11:59–65. 10.2147/OTT.S147715 29317834 PMC5743191

[53] Oh SE, Seo JE, An JY, Lee JH, Sohn TS, Bae JM, Kim S, Choi MG. Prognostic Impact of Increased Perioperative Platelet Count in Gastric Cancer Patients. J Surg Res. 2019; 242:296–303. 10.1016/j.jss.2019.04.052 31125843

[54] Giannakeas V, Kotsopoulos J, Brooks JD, Cheung MC, Rosella L, Lipscombe L, Akbari MR, Austin PC, Narod SA. Platelet Count and Survival after Cancer. Cancers (Basel). 2022; 14:549. 10.3390/cancers14030549 35158817 PMC8833779

[55] Giannakeas V, Kotsopoulos J, Cheung MC, Rosella L, Brooks JD, Lipscombe L, Akbari MR, Austin PC, Narod SA. Analysis of Platelet Count and New Cancer Diagnosis Over a 10-Year Period. JAMA Netw Open. 2022; 5:e2141633. 10.1001/jamanetworkopen.2021.41633 35015064 PMC8753503

[56] Giannakeas V. Trends in platelet count among cancer patients. Exp Hematol Oncol. 2022; 11:16. 10.1186/s40164-022-00272-3 35331331 PMC8944120

[57] Mammadova-Bach E, Gil-Pulido J, Sarukhanyan E, Burkard P, Shityakov S, Schonhart C, Stegner D, Remer K, Nurden P, Nurden AT, Dandekar T, Nehez L, Dank M, et al. Platelet glycoprotein VI promotes metastasis through interaction with cancer cell-derived galectin-3. Blood. 2020; 135:1146–60. 10.1182/blood.2019002649 32040544

[58] Neuwelt AJ, Kimball AK, Johnson AM, Arnold BW, Bullock BL, Kaspar RE, Kleczko EK, Kwak JW, Wu MH, Heasley LE, Doebele RC, Li HY, Nemenoff RA, Clambey ET. Cancer cell-intrinsic expression of MHC II in lung cancer cell lines is actively restricted by MEK/ERK signaling and epigenetic mechanisms. J Immunother Cancer. 2020; 8:e000441. 10.1136/jitc-2019-000441 32312906 PMC7204826

[59] Tang Q, Zhang H, Kong M, Mao X, Cao X. Hub genes and key pathways of non-small lung cancer identified using bioinformatics. Oncol Lett. 2018; 16:2344–54. 10.3892/ol.2018.8882 30008938 PMC6036325

[60] Govindan R, Ding L, Griffith M, Subramanian J, Dees ND, Kanchi KL, Maher CA, Fulton R, Fulton L, Wallis J, Chen K, Walker J, McDonald S, et al. Genomic landscape of non-small cell lung cancer in smokers and never-smokers. Cell. 2012; 150:1121–34. 10.1016/j.cell.2012.08.024 22980976 PMC3656590

[61] Yang F, Zhang S, Meng Q, Zhou F, Pan B, Liu F, Yu Y. CXCR1 correlates to poor outcomes of EGFR-TKI against advanced non-small cell lung cancer by activating chemokine and JAK/STAT pathway. Pulm Pharmacol Ther. 2021; 67:102001. 10.1016/j.pupt.2021.102001 33582208

[62] Koupenova M, Livada AC, Morrell CN. Platelet and Megakaryocyte Roles in Innate and Adaptive Immunity. Circ Res. 2022; 130:288–308. 10.1161/CIRCRESAHA.121.319821 35050690 PMC8852355

[63] Ma Z, Yu YR, Badea CT, Kovacs JJ, Xiong X, Comhair S, Piantadosi CA, Rajagopal S. Vascular Endothelial Growth Factor Receptor 3 Regulates Endothelial Function Through β-Arrestin 1. Circulation. 2019; 139:1629–42. 10.1161/CIRCULATIONAHA.118.034961 30586762 PMC6433500

[64] Ma H, Wang L, Zhang T, Shen H, Du J. Loss of β-arrestin1 expression predicts unfavorable prognosis for non-small cell lung cancer patients. Tumour Biol. 2016; 37:1341–7. 10.1007/s13277-015-3886-0 26293896

[65] El-Khoury V, Béland M, Schritz A, Kim SY, Nazarov PV, Gaboury L, Sertamo K, Bernardin F, Batutu R, Antunes L, Bennett CW, Faÿs F, Berchem G, Kim YJ. Identification of beta-arrestin-1 as a diagnostic biomarker in lung cancer. Br J Cancer. 2018; 119:580–90. 10.1038/s41416-018-0200-0 30078843 PMC6162208

[66] Fu L, Deng R, Huang Y, Yang X, Jiang N, Zhou J, Lin C, Chen S, Wu L, Cui Q, Yun J. DGKA interacts with SRC/FAK to promote the metastasis of non-small cell lung cancer. Cancer Lett. 2022; 532:215585. 10.1016/j.canlet.2022.215585 35131384

[67] Khalaji A, Haddad S, Yazdani Y, Moslemi M, Alizadeh L, Baradaran B. A bioinformatics-based study on the Cisplatin-resistant lung cancer cells; what are the orchestrators of this phenom? Gene. 2022; 834:146668. 10.1016/j.gene.2022.146668 35690284

[68] Wang H, Meyer CA, Fei T, Wang G, Zhang F, Liu XS. A systematic approach identifies FOXA1 as a key factor in the loss of epithelial traits during the epithelial-to-mesenchymal transition in lung cancer. BMC Genomics. 2013; 14:680. 10.1186/1471-2164-14-680 24093963 PMC3852829

[69] Zhang X, Wang F, Huang Y, Ke K, Zhao B, Chen L, Liao N, Wang L, Li Q, Liu X, Wang Y, Liu J. FGG promotes migration and invasion in hepatocellular carcinoma cells through activating epithelial to mesenchymal transition. Cancer Manag Res. 2019; 11:1653–65. 10.2147/CMAR.S188248 30863175 PMC6389006

[70] Wang M, Zhang G, Zhang Y, Cui X, Wang S, Gao S, Wang Y, Liu Y, Bae JH, Yang WH, Qi LS, Wang L, Liu R. Fibrinogen Alpha Chain Knockout Promotes Tumor Growth and Metastasis through Integrin-AKT Signaling Pathway in Lung Cancer. Mol Cancer Res. 2020; 18:943–54. 10.1158/1541-7786.MCR-19-1033 32205365

[71] Kuang M, Peng Y, Tao X, Zhou Z, Mao H, Zhuge L, Sun Y, Zhang H. FGB and FGG derived from plasma exosomes as potential biomarkers to distinguish benign from malignant pulmonary nodules. Clin Exp Med. 2019; 19:557–64. 10.1007/s10238-019-00581-8 31576477

[72] Zhang W, Gao Z, Zeng G, Xie H, Liu J, Liu N, Wang G. Clinical significance of urinary plasminogen and fibrinogen gamma chain as novel potential diagnostic markers for non-small-cell lung cancer. Clin Chim Acta. 2020; 502:55–65. 10.1016/j.cca.2019.11.022 31821791

[73] Liu Y, Song Y, Cao M, Fan W, Cui Y, Cui Y, Zhan Y, Gu R, Tian F, Zhang S, Cai L, Xing Y. A novel EHD1/CD44/Hippo/SP1 positive feedback loop potentiates stemness and metastasis in lung adenocarcinoma. Clin Transl Med. 2022; 12:e836. 10.1002/ctm2.836 35485206 PMC9786223

[74] Gao J, Meng Q, Zhao Y, Chen X, Cai L. EHD1 confers resistance to cisplatin in non-small cell lung cancer by regulating intracellular cisplatin concentrations. BMC Cancer. 2016; 16:470. 10.1186/s12885-016-2527-3 27411790 PMC4944258

[75] Yuan R, Huang Y, Chan L, He D, Chen T. Engineering EHD1-Targeted Natural Borneol Nanoemulsion Potentiates Therapeutic Efficacy of Gefitinib against Nonsmall Lung Cancer. ACS Appl Mater Interfaces. 2020; 12:45714–27. 10.1021/acsami.0c08069 32927941

[76] Saini A, Chandra KB, Kumar V, Mathur SR, Sharma JB, Kumar S, Yadav S. Analysis of Multimerin 1 (MMRN1) expression in ovarian cancer. Mol Biol Rep. 2020; 47:9459–68. 10.1007/s11033-020-06027-9 33263168

[77] Laszlo GS, Alonzo TA, Gudgeon CJ, Harrington KH, Gerbing RB, Wang YC, Ries RE, Raimondi SC, Hirsch BA, Gamis AS, Meshinchi S, Walter RB. Multimerin-1 (MMRN1) as Novel Adverse Marker in Pediatric Acute Myeloid Leukemia: A Report from the Children's Oncology Group. Clin Cancer Res. 2015; 21:3187–95. 10.1158/1078-0432.CCR-14-2684 25825478 PMC4506237

[78] Li C, Liu T, Liu Y, Zhang J, Zuo D. Prognostic value of tumour microenvironment-related genes by TCGA database in rectal cancer. J Cell Mol Med. 2021; 25:5811–22. 10.1111/jcmm.16547 33949771 PMC8184694

[79] Saini A, Kumar V, Tomar AK, Sharma A, Yadav S. Multimerin 1 aids in the progression of ovarian cancer possibly via modulation of DNA damage response and repair pathways. Mol Cell Biochem. 2023. [Epub ahead of print]. 10.1007/s11010-023-04668-5 36723821

[80] Välk K, Vooder T, Kolde R, Reintam MA, Petzold C, Vilo J, Metspalu A. Gene expression profiles of non-small cell lung cancer: survival prediction and new biomarkers. Oncology. 2010; 79:283–92. 10.1159/000322116 21412013

[81] Lazer G, Katzav S. Guanine nucleotide exchange factors for RhoGTPases: good therapeutic targets for cancer therapy? Cell Signal. 2011; 23:969–79. 10.1016/j.cellsig.2010.10.022 21044680

[82] Bae JY, Choi KU, Kim A, Lee SJ, Kim K, Kim JY, Lee IS, Chung SH, Kim JI. Evaluation of immune-biomarker expression in high-grade soft-tissue sarcoma: HLA-DQA1 expression as a prognostic marker. Exp Ther Med. 2020; 20:107. 10.3892/etm.2020.9225 32989386 PMC7517476

[83] Zeng RJ, Xie WJ, Zheng CW, Chen WX, Wang SM, Li Z, Cheng CB, Zou HY, Xu LY, Li EM. Role of Rho guanine nucleotide exchange factors in non-small cell lung cancer. Bioengineered. 2021; 12:11169–87. 10.1080/21655979.2021.2006519 34783629 PMC8810164

[84] Zhang Q, Liu S, Wang H, Xiao K, Lu J, Chen S, Huang M, Xie R, Lin T, Chen X. ETV4 Mediated Tumor-Associated Neutrophil Infiltration Facilitates Lymphangiogenesis and Lymphatic Metastasis of Bladder Cancer. Adv Sci (Weinh). 2023; 10:e2205613. 10.1002/advs.202205613 36670069 PMC10104629

[85] Huang M, Dong W, Xie R, Wu J, Su Q, Li W, Yao K, Chen Y, Zhou Q, Zhang Q, Li W, Cheng L, Peng S, et al. HSF1 facilitates the multistep process of lymphatic metastasis in bladder cancer via a novel PRMT5-WDR5-dependent transcriptional program. Cancer Commun (Lond). 2022; 42:447–70. 10.1002/cac2.12284 35434944 PMC9118058

[86] Cascone T, Fradette J, Pradhan M, Gibbons DL. Tumor Immunology and Immunotherapy of Non-Small-Cell Lung Cancer. Cold Spring Harb Perspect Med. 2022; 12:a037895. 10.1101/cshperspect.a037895 34580079 PMC8957639

