# Supplementary material for: Characterization of platelet-related genes and constructing signature combined with immune-related genes for predicting outcomes and immunotherapy response in lung squamous cell carcinoma
Source: Aging (Albany NY). 2023 Jul 20;15(14):6969–92. doi: 10.18632/aging.204886 (PMC10415560; doi:10.18632/aging.204886)
Supplement: Supplementary Figure 1 [file aging-15-204886-s001.pdf]

SUPPLEMENTARY FIGURE

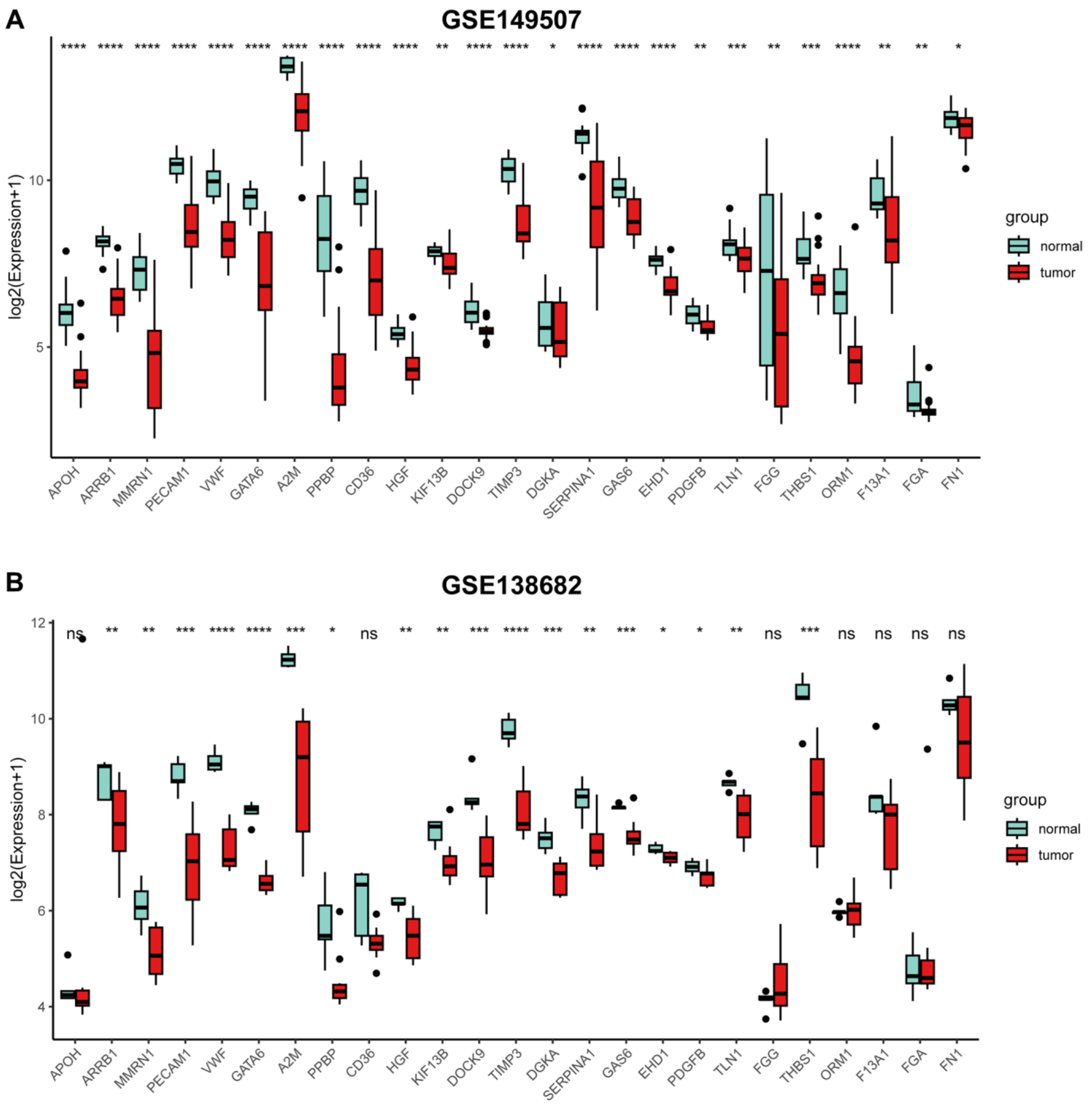

Supplementary Figure 1. The expression analysis of 25 PRGs in public database. (GSE149507 and GSE138682). (**A**, **B**) Boxplot showed the expression of 25 PRGs in lung cancer compared to normal tissues in two GEO cohorts. $^{*}P < 0.05$, $^{**}P < 0.01$, $^{***}P < 0.001$, $^{****}P < 0.0001$ and ns represents not significant.
