# Supplementary material for: Characterization of platelet-related genes and constructing signature combined with immune-related genes for predicting outcomes and immunotherapy response in lung squamous cell carcinoma
Source: Aging (Albany NY). 2023 Jul 20;15(14):6969–92. doi: 10.18632/aging.204886 (PMC10415560; doi:10.18632/aging.204886)
Supplement: Supplementary Table 1 [file aging-15-204886-s002.pdf]

SUPPLEMENTARY TABLE

Supplementary Table 1. Basic information of datasets included in this study.

Accession number/ Source	Platform	Sample	
		Tumor	Normal
TCGA-LUSC	RNA-seq	489	49
GSE3141	Affymetrix Human Genome U133 Plus 2.0 Array	61	0
GSE12472	Agilent-012391 Whole Human Genome Oligo Microarray G4112A	35	0
GSE30219	Affymetrix Human Genome U133 Plus 2.0 Array	110	0
GSE157011	Affymetrix Human Genome U133 Plus 2.0 Array	484	0
GSE138682	Affymetrix Human Genome U133 Plus 2.0 Array	5	5
GSE149507	Affymetrix Human Genome U133 Plus 2.0 Array	18	18
GSE78220	Illumina HiSeq 2000	28	0
GSE176307	Ion Torrent S5 XL	90	0
GSE135222	Illumina HiSeq 2500	27	0
E-MTAB-6149	10x Genomics	5	0
